# Mesenchymal Stem or Stromal Cells from Amnion and Umbilical Cord Tissue and Their Potential for Clinical Applications

**DOI:** 10.3390/cells1041061

**Published:** 2012-11-12

**Authors:** Andrea Lindenmair, Tim Hatlapatka, Gregor Kollwig, Simone Hennerbichler, Christian Gabriel, Susanne Wolbank, Heinz Redl, Cornelia Kasper

**Affiliations:** 1 Ludwig Boltzmann Institute for Experimental and Clinical Traumatology, AUVA Research Center, Vienna/Linz 1200, Austria; Email: Andrea.Lindenmair@o.roteskreuz.at (A.L.); Susanne.Wolbank@TRAUMA.LBG.AC.AT (S.W.); Office@TRAUMA.LBG.AC.AT (H.R.); 2 Austrian Cluster for Tissue Regeneration, Austria; Email: Simone.Hennerbichler@o.roteskreuz.at (S.H.); Christian.Gabriel@o.roteskreuz.at (C.G.); 3 Department of Biotechnology, University of Natural Resources and Life Sciences, Vienna 1190, Austria; Email: Tim.Hatlapatka@boku.ac.at (T.H.); Gregor.Kollwig@boku.ac.at (G.K.); 4 Red Cross Blood Transfusion Service of Upper Austria, Linz 4020, Austria

**Keywords:** MSC, mesenchymal stem cell, mesenchymal stromal cell, amnion, umbilical cord, clinical applications

## Abstract

Mesenchymal stem or stromal cells (MSC) have proven to offer great promise for cell-based therapies and tissue engineering applications, as these cells are capable of extensive self-renewal and display a multilineage differentiation potential. Furthermore, MSC were shown to exhibit immunomodulatory properties and display supportive functions through parakrine effects. Besides bone marrow (BM), still today the most common source of MSC, these cells were found to be present in a variety of postnatal and extraembryonic tissues and organs as well as in a large variety of fetal tissues. Over the last decade, the human umbilical cord and human amnion have been found to be a rich and valuable source of MSC that is bio-equivalent to BM-MSC. Since these tissues are discarded after birth, the cells are easily accessible without ethical concerns.

## 1. Introduction

Mesenchymal cells derived from amniotic membrane have been referred to in various ways by different research groups, including human amnion/amniotic mesenchymal stromal cells (hAMSC[s]), amniotic membrane mesenchymal stem cells (AM-MSC), amniotic membrane-human mesenchymal stromal cells (AM-hMSC), amnion-derived MSC, amniotic mesenchymal fibroblasts, human amnion stromal cells (hASC), human amniotic mesenchymal tissue cells (AMTC), human amniotic mesenchymal cells (HAMc), mesenchymal cells derived from human amniotic membrane (MC-HAM), human amniotic mesenchymal stem cells (hAMs), human amniotic membrane-derived mesenchymal cells (hAMCs or hAM-MSC) and human amnion-derived fibroblast-like cells (HADFIL) [1,2,3,4,5,6,7,8,9,10,11,12,13,14,15,16,17,18,19,20,21,22,23,24,25]. During the first meeting of the “International Placenta Stem Cell Society” (IPLASS) in 2007, it was agreed that the nomenclature of these cells should be unified as “human amniotic mesenchymal stromal cells” (hAMSC) [19].

hAMSC are derived from the extraembryonic mesoderm [19] and are found dispersed in the collagenous stroma underlying the epithelial monolayer of the amniotic membrane [2,16].

The nomenclature of umbilical cord (UC)-derived MSC has not been unified yet and the cells have been referred to in many ways, mainly depending on from which compartment of the cord the cells have been isolated. The UC usually comprises two arteries and a vein, which are immersed within a mucoid connective tissue, the so-called Wharton’s jelly (WJ), and are enclosed by a simple amniotic epithelium. At least four separate regions were found to contain mesenchymal cells. Thus, MSC could be isolated from the subendothelium [26,27,28], the WJ [29,30,31,32,33,34,35,36,37,38,39], the perivascular region [40,41], and the umbilical cord blood [42,43,44,45,46]. Parts of this review will focus on UC tissue-derived MSC which will be referred to as UC-MSC.

## 2. Isolation, Expansion and Characterization of hAMSC

Amniotic membrane is mechanically peeled off the chorionic membrane by blunt dissection, washed several times in a buffered solution and cut into small pieces. In most cases, hAMSC are obtained in subsequent enzymatic digestions. After complete removal of human amniotic epithelial cells (hAEC) by trypsin digestion, hAMSC are digested with various types and concentrations of collagenase (0.75–2 mg/mL) with or without adding DNase (20–75 μg/mL) [8,11,17,19,23,47,48,49,50,51,52]. Lisi *et al.* [14] have additionally added trypsin; Tawagawa *et al.* [53] have supplemented dispase and papain. Some groups have published that pure fractions of hAMSC can be obtained without previous isolation of hAEC treatment [10,49,52,54,55]. Due to the varying concentrations of collagenase used by the different groups, also the applied incubation times vary between 30 min and up to 3 h. For gaining both hAMSC and hAEC also reversed isolation protocols (hAEC after hAMSC) are published [52,56]. 

According to Parolini *et al.* [19], one single amnion should theoretically contain 5 × 10^8^ hAMSC. Typically, one gram tissue yields in about 1–2 × 10^6^ hAMSC [2,19]. Media used for expansion are usually composed of a basal medium, supplemented with fetal calf serum, antibiotics and antimycotics. The detailed compositions and some further used supplements that have been published for hAMSC expansion are listed in Table 1

**Table 1:** Media used for expansion of human amnion/amniotic mesenchymal stromal cells (hAMSC); Abbreviations: FCS fetal calf serum, DMEM Dulbecco’s modified Eagles’s medium, EGF epidermal growth factor, M199 medium 199, b-ME beta-mercaptoethanol, NEAA nonessential amino acid, LIF leukemia inhibitory factor, MEM minimal essential medium eagle.

	basal	% FCS	further supplements
Bilic [47,57]	DMEM:Ham'sF12 1:1	10	50 ng/mL EGF, 2.5 μg/mL insulin, 5 μg/mL transferrin, 0.1 ng/mL tri-iodothyronine
Bilic [2]	DMEM:Ham'sF12 1:1	10	for some experiments 10 ng/mL EGF
Diaz-Prado [4]	DMEM	20	
In't Anker [8]	M199	10	20 μg/mL EGF, 8 U/mL heparin
Kang [10]	alphaMEM	10	
Kim [11]	DMEM	10	3.7 mg/mL sodium bicarbonate
König [12]	DMEM	15	
Lisi [14]	DMEM	10	10 ng/mL EGF, 55 μM b-ME
Paracchini [18]	DMEM	10	1% sodium pyruvate, 1% NEAA, 55 μM b-ME, 10 ng/mL EGF
Stadler [21]	DMEM	10	1% NEAA, 55 μM 2-mercaptoethanol, 1 mM sodium pyruvate
Sudo [56]	alphaMEM	10	without any further supplements or 10 ng/mL EGF or 10 ng/mL EGF + 10^5^ U/mL LIF
Tamagawa [53]	alphaMEM	10	10 ng/mL EGF, 10 ng/mL LIF
Whittle [54]	DMEM	10	
Zhao [25]	DMEM	10	

Some groups cultivate the cells in endothelial growth medium-2 (EGM-2) [12,13,21,55], which is a 2% serum medium supplemented with hydrocortisone, heparin, ascorbic acid, gentamicin sulfate and various growth factors (insulin-like growth factor (IGF), vascular endothelial growth factor (VEGF), epidermal growth factor (EGF) and fibroblast growth factor FGF)).

One characteristic property of mesenchymal stem cells such as hAMSC is their plastic adherence. However, some groups published coating of the culture dishes with gelatin or fibronectin [8,12]. To remove non-adherent cells, medium is removed after a time of two h [18] up to seven days [1,8,14] after cell seeding. After reaching confluence of 70%–100%, cells are usually detached with trypsin (0.05% or 0.25%) with or without EDTA (0.02%) [1,4,8,11,14,18,21,53,56]. Alternatively, also application of accutase is reported [12]. There is a great variability regarding seeding density, reaching from 1 × 10^3^ c/cm^2^ [1] up to 1.27 × 10^5^ c/cm^2^ [56]. 

Expansion of hAMSC is possible for at least five passages without any morphological alterations [1,2,9,14,19]. Some groups have even kept the cells in culture for 15 to 20 passages before reaching senescence [53,57]. 

hAMSC show fibroblast-like cell morphology, being spindle-shaped [4,11,19,53]. Regarding the ability to form colonies, there are differing reports. Soncini *et al.* [52] and Kim *et al.* [11], have shown clonal colony formation, whereas Bilic *et al.* [2] did not detect any clonal outgrowth, supposedly due to the lack of telomerase reverse transcriptase (TERT).

Analyzing the surface antigen profile by flow cytometry, polymerase chain reaction or immunocytochemistry staining, hAMSC are found to express the mesenchymal markers CD73, CD90 as well as CD105 and are further positive for CD10, CD13, CD29, CD44, CD49c, CD49d, CD49e, CD54, CD140b, CD166, CD349, STRO-1 and HLA-ABC [1,2,6,8,12,14,19,20,50,55,58]. Weak expression has been reported for CD271 [19,20] and CD117 (ckit) [2,4], in one case only being detected using PCR [58]. The hematopoietic markers CD34 and CD45, the monocyte marker CD14, the endothelial markers CD31 and CD133, as well as CD3 and CD11 are not expressed on hAMSC [1,2,5,6,8,12,14,19,50]. HLA-DR is reported to be absent or expressed at very low levels [1,2,6,8,12,14,49,55]. Paracchini *et al.* [18] analyzed low levels of EpCAM and CD49f in fresh hAMSC cultures, but these markers were rapidly decreasing during expansion. Immunofluorescence staining of amniotic membrane did not reveal SSEA-3 and SSEA-4 [59], however, surface expression of these markers on hAMSC is reported by several groups [4,6,11,18,19,58,59,60]. Furthermore, RNA levels of the transcription factor Oct-4 are reported [2,6,55,58] to be even higher than in bone marrow derived mesenchymal stem cells [1]. Transmission electron microscopy of hAMSC has revealed ultrastructural characteristics of mesenchymal as well as epithelial cells, showing a sign of multipotentiality [61].

## 3. Isolation, Expansion and Characterization of UC-MSC

**Figure 1:**
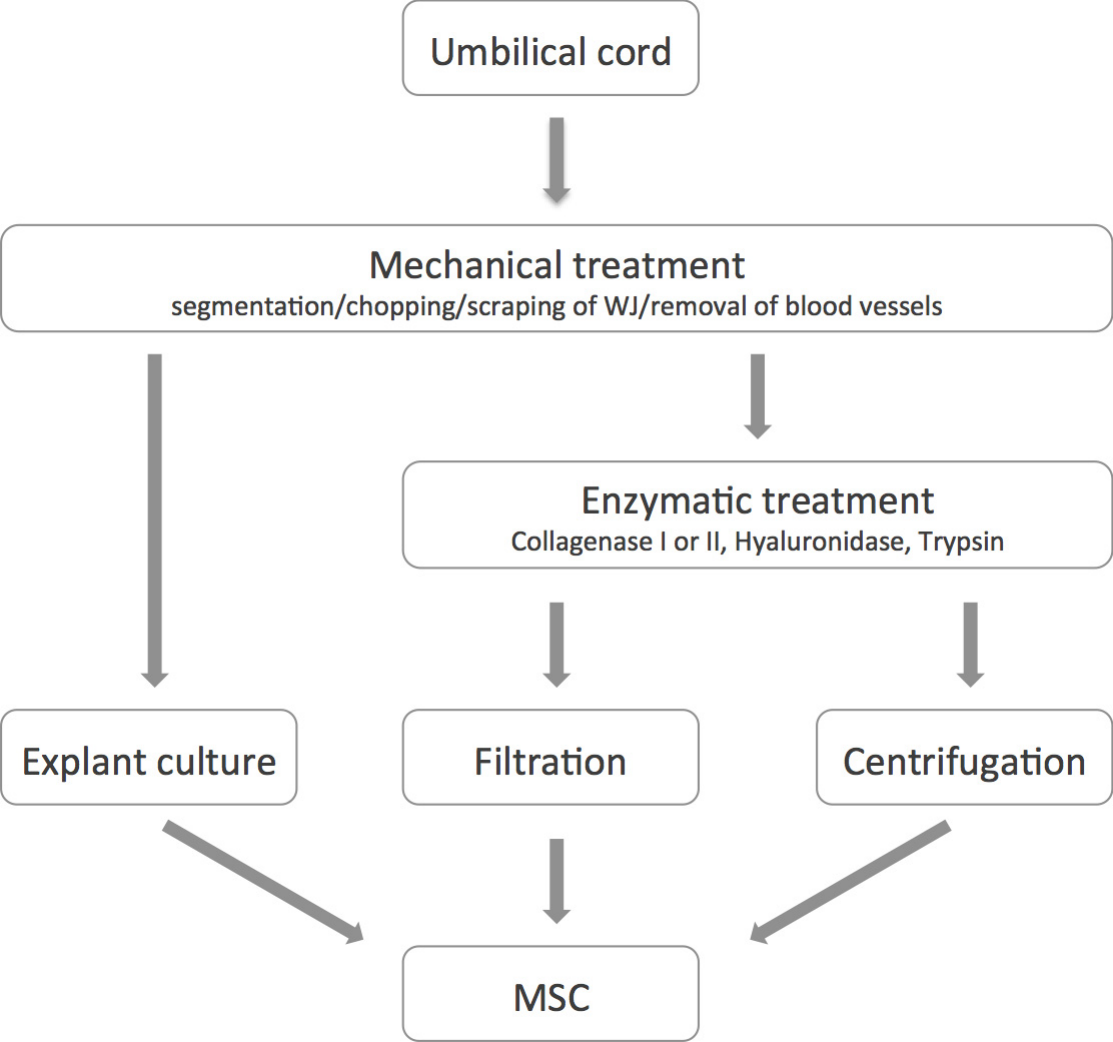
Different techniques used during isolation of mesenchymal stem or stromal cells (MSC) from umbilical cord (UC) tissue. Basically enzymatic digestion or explant culture approaches are used.

Besides the umbilical cord blood the umbilical cord tissue was also found to be a rich and valuable source of MSC. For the isolation of the cells many protocols have been proposed mainly depending on from which compartment the cells should be isolated. An overview of different techniques applied during isolation is given in Figure 1. Basically the isolation procedure starts with a mechanical treatment of the tissue. This may contain a segmentation of the cord, chopping into small tissue pieces or scraping of the Wharton’s Jelly. Often the umbilical arteries and vein are removed and discarded before further processing [31,33,39,62] but the perivascular regions, the vessels and the sub endothelium of the vein can also serve as a source of MSC [26,27,28,40,41,63]. Most of the protocols contain steps of enzymatic digestion of the tissue with several enzymes (e.g., Collagenase I or II, Hyaluronidase and Trypsin) followed by filtration and or centrifugation steps [31,33,39,62,64,65,66,67] but explant culture approaches are also described [68,69,70,71]. By cutting down the tissue into approximately 0.5 cm^3^ large pieces and incubating them in appropriate culture media at 37 °C and a humidified atmosphere with 5% CO_2_, adherent cells will start to grow out of the tissue after approximately 10 days resulting in a confluent culture after two weeks [70].

Freshly isolated UC-derived MSC are mainly fibroblast-like spindle-shaped cells. Although isolated from different compartments of the cord by several isolation techniques, all UC-derived cells are found to meet the minimal criteria for defining multipotent mesenchymal stromal cells as proposed by the ISCT. Thus, besides growing adherent to plastic and exhibiting a multi-lineage differentiation potential, the cells express CD105, CD73 and CD90, and lack the expression of CD45, CD34, CD14 or CD11b, CD79 alpha or CD19 and HLA-DR. An overview of further reported intra- and extra-cellular markers expressed by UC-derived MSC is given in [72].

**Figure 2:**
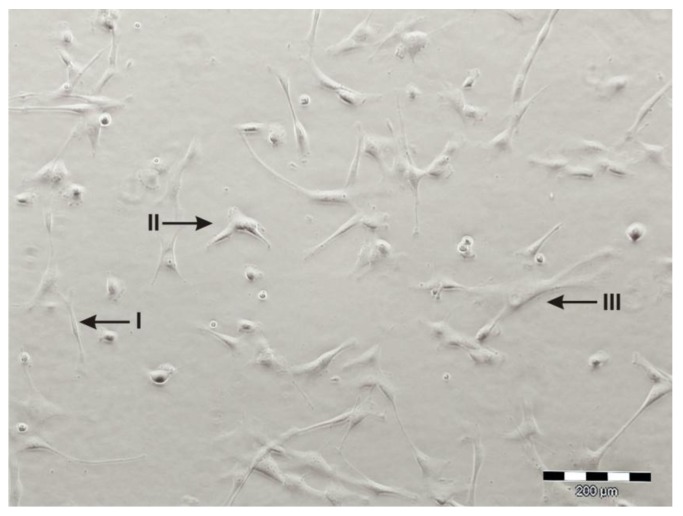
Primary UC-derived cell cultures can contain broad cell size distributions (I < II < III).

However, some groups observed varying phenotypes among UC-derived primary cells (see Figure 2). The cultures displayed a broad cell size distribution and when using counterflow centrifugal elutriation (CCE) the cells could be separated according to their size leading to MSC subpopulations that, although sharing the same immunophenotype, displayed significant differences in cell growth and biochemical marker expression [73]. 

Additionally, when cells isolated from the Wharton’s Jelly were compared to UC arterial- and venous-derived cells significant differences could be observed with regard to proliferative and osteogenic differentiation potential [63]. These results demonstrate the demand for further investigations concerning UC-MSC from different UC-compartments and MSC sub-populations but also raise the question whether the proposed minimal criteria are still sufficiently defined to identify MSC not only from UC but other tissues. In this context, several groups have proposed new surface antigens as universal markers for the identification of MSC from bone marrow and other tissues (reviewed in [74]) such as CD271 [75,76], MSCA-1 [75], SSEA-4 [77,78], and the neural ganglioside GD2 [79]. To our knowledge MSCA-1 expression in UC-MSC cultures have not been investigated yet. Additionally UC-derived cell populations were found to be negative for CD271 surface antigen expression but express SSEA-4 [63,68]. Furthermore, UC-tissue was shown to harbor a subset of GD2^+^ cells that exhibit a high clonogenicity as well as proliferation capacity but also a significantly stronger multi-differentiation potential than GD2^−^ cells, indicating GD2 to be a potential marker useful for the isolation of multipotent MSC from UC-tissue [80]. 

## 4. Differentiation Potential of hAMSC

Besides differentiating into the typical mesenchymal lineages—osteogenic, chondrogenic, adipogenic—hAMSC are also capable to differentiate into cells of all three germ layers: ectoderm (neural, glial), mesoderm (skeletal muscle, endothelial, cardiomyocytic) and endoderm (pancreatic, hepatic).

The osteogenic lineage was one of the first being demonstrated for hAMSC [8,50]. Osteogenic stimulatory media that have been applied (listed in detail in Table 2) are at least supplemented with beta-glycerophosphate, dexamethasone and ascorbat-2-phosphate or ascorbic acid. Mineral deposition upon osteogenic induction has been demonstrated by von Kossa and Alizarinred S staining [1,2,4,8,10,18,21,24,56,81]. Further, alkaline phosphatase activity, as well as staining for alkaline phosphatase and osteocalcin, have been used to proof osteogenic differentiation [2,8,21,56,57,81]. Differentiation efficiency has been further evaluated by PCR for specific markers involved in osteogenesis: the early marker RUNX2, ALP, followed by later markers BGLAP, BMPR1B and BMPR2 [21,24,81]. Stadler *et al.* [21] have demonstrated the impact of four different *in vitro* protocols for osteogenic differentiation – the media according to Pittenger *et al.* [82], In’t Anker *et al.* [8] and Portmann-Lanz *et al.* [50] as well as hMSC Mesenchymal Stem Cell Osteogenic Differentiation Medium (OKit, Lonza). Moreover, hAMSC grown on microcarriers have been demonstrated to differentiate into osteogenic lineage with improved potency [81]. These hAMSC-laden microcarriers were further used for fabricating a macroscopic bone construct in a cylindrical perfusion culture chamber.

Differentiation towards the adipogenic lineage has been achieved with the media listed in Table 2. The formation of multivacuolated cells was proven by Oil Red O staining [1,4,10,18,21,24,56,57] and the induction of mRNA levels of the adipo-specific genes—PPARgamma, leptin, lipoprotein lipase and fatty acid binding protein aP2—were demonstrated [24,56]. Comparing three different induction media for adipogenic differentiation—the media according to Pittenger *et al.* [82], In’t Anker *et al.* [8] as well as Portmann-Lanz *et al.* [50]—Stadler *et al.* [21] have reported two of four donors being capable of adipogenic conversion. Paracchini *et al.* [18] observed morphological changes as well as formation of lipid droplets starting from one week after induction. However, due to considerable donor variability and potentially also due to the use of different protocols, only few hAMSC donors have been shown to be capable of converting into adipocyte-like cells [2,21,24,56]. 

Chondrogenic differentiation has mostly been demonstrated in pellet cultures using between 2.5 × 10^5^ and 5.0 × 10^5^ cells per pellet [1,4,10,56]. For induction of chondrogenic differentiation of hAMSC, transforming growth factor-beta (TGF-b) was supplemented in all 3D-cultures. Detailed media compositions are listed in Table 2. hAMSC differentiated towards the chondrogenic lineage produced abundant extracellular matrix visualized by toluidin or alician blue staining. Further they stained positive for collagen type II and expressed mRNA of SOX-9, SOX-5, SOX-6, BMP-2, BMP-4, BMP receptor (BMPR)-1A, BMPR-1B, BMPR-2, COMP, osteocalcin and aggrecan [1,4,10,23,50,56]. Although 3D-culture may be preferential to support chondrogenic differentiation chondrogenic differentiation has even been demonstrated in a confluent monolayer [23,50].

Myogenic differentiation, another mesodermal lineage, has first been proven by Portmann-Lanz *et al.* [50], showing expression of the transcription factor MyoD as well as skeletal muscle myosin heavy chain in 15%–35% of induced hAMSC. Confirming these results, Alviano *et al.* [1] evaluated mRNA expression levels of MyoD and Myogenin after 7 and 14 days, respectively. Immunocytochemical staining further visualized desmin-positive cells after 3 weeks. 

Alviano *et al.* [1] demonstrated also that hAMSC differentiate towards the angiogenic lineage in the matrigel assay, showing increased expression of FLT-1, KDR and ICAM-1. Adding VEGR even induced expression of CD34 and von Willebrand factor (vWF). However, there are controversial reports regarding angiogenic differentiation. Kim *et al.* [83] confirmed endothelial differentiation of hAMSC being cultured in EGM-2 demonstrating FLT-1, KDR, Tie-2 and vWF. König *et al.* [12] demonstrated morphological changes towards endothelial-like appearance, uptake of acetylated low-density lipoprotein as well as formation of endothelial-like structures in the matrigel assay. However, they have not found hAMSC positive for vWF and VE-cadherin and they assumed that hAMSC resisted differentiating into mature endothelial cells. They further analyzed angiogenic differentiation of hAMSC using microarray analysis. Interestingly, pro-angiogenic factors (tenascinC, Tie-2, VEGF-A, CD146, FGF-2) were found to be downregulated, whereas serpin F1, sprout 1 and angioarrestin were upregulated. Supplementing an endothelial cell line culture with the supernatants of endothelially-induced hAMSC supplied network-like structures in the matrigel assay. Kim *et al.* [83] confirmed these paracrine effects of hAMSC using conditioned media of induced hAMSC in a matrigel-assay with HUVEC. 

Cardiomyogenic differentiation of hAMSC has been demonstrated [25,58] and the differentiated cells have already been combined with a scaffold [14]. Undifferentiated hAMSC inherently express cardiac-specific genes and transcription factors: Myosin light chain-2a (MLC-2a), MLC-2v, GATA-4, cardiac troponin (cTn) T, cTnI and the cardiac-specific ion channel genes alpha1c and Kv4.3 [25]. After stimulation with bFGF and activin A, also the cardiac-specific transcription factor Nkx2.5 and human atrial natriuretic peptide were expressed. Co-culturing hAMSC with rat heart explants revealed integration and transdifferentiation of hAMSC into cardiomyocyte-like cells. Tsuji *et al.* [58] could increase cardiomyogenic transdifferentiation pretreating hAMSC with interleukin-10 or progesterone and even demonstrated spontaneously beating cells derived from hAMSC when co-cultured with fetal murine cardiomyocytes. Moreover, hAMSC stimulated with S-nitroso-N-acetylpenicillamine towards the cardiomyogenic lineage have been combined with a s-IPN PEtU-PDMS/fibrin scaffold, demonstrating increased cardiac and vascular markers [14].

Due to their multipotency, hAMSC are also able to differentiate into endodermal lineages including hepatocyte-like and pancreatic cells. Undifferentiated hAMSC already express albumin, alpha-fetoprotein, cytokeratin (CK) 18 and alpha1-antitrypsin [53]. Following hepatic induction the expression of these factors was increased and additionally storage of glycogen was clearly detected. Another hepatocyte differentiation protocol was recently published by Paracchini *et al.* [18]. Beside weak expression of alpha1-antitrypsin, increased numbers of cells expressed CK7, albumin and CK19. Furthermore, functionality of cytochrome P450-dependent mixed function oxidase was demonstrated. 

Pancreatic differentiation of hAMSC was first demonstrated by Wei *et al.* [84] although they reported higher differentiation efficiency when using hAEC. Tamagawa *et al.* [22] designed a three step procedure (details listed in Table 2) demonstrating successful pancreatic differentiation of hAMSC. Morphology changed to rounder epithelial-like cells and many islet-like clusters were observed during the second step applying matrigel. Initial mRNA expression of CK19, Pdx-1 and Nkx2.2 were increased and insulin, glucagon, Isl-1, GLUT-2, GCK, PAX-6, Nkx6-1 and NeuroD were detected after induction. Immunostaining even revealed the proteins insulin, glucagon and somatostatin. Kim *et al.* [11] recently published the generation of functional insulin-secreting cells derived from hAMSC in a one step procedure. They could confirm that undifferentiated hAMSC already showed low levels of expression of mRNA of pancreatic cell-related genes (NEUROD1, GLUT1, PC2 and GCK). Upon induction, hAMSC showed increased expression of INS, PDX, Nkx6-1, NEUROG3, ISL1, NEUROD1, GLUT1, PC1/3, PC2, GCK, PPY, SST and GCG. Moreover, induced cells secreted insulin as well as c-peptide in a glucose-dependent manner. 

hAMSC are further capable to differentiate towards cells derived from the ectodermal lineages. Portmann-Lanz *et al.* [50] differentiated hAMSC towards the neurogenic lineage revealing subpopulations stained positive for CD133, nestin and neurofilament 200. Phenotypes of neuroglial progenitor cells were observed applying the protocol of Sakuragawa *et al.* [51]. Unstimulated hAMSC, which express mRNA of the typical neural markers nestin and Musashi1, were induced to increase expression of these genes as well as significantly upregulate beta-tubulin isotype III (Tuj1) and neurofilament-medium (NF-M). Furthermore, also the number of glial fibrillary acidic protein (GFAP) positive cells increased. Tamagawa *et al.* [85] have demonstrated morphological changes—retracted bodies, long processes, neuron network-like structures—and expression of neuron specific enolase, NF-M, TUJ1, GFAP as well as myelin basic protein after neural induction. Manochantr *et al.* [57] evaluated a commercially available neural medium—AdvancesSTEM Neural Differentiation Medium (HyClone)—demonstrating sharp, elongated bi-or-tripolar cells expressing MAP-2, TUJ1 and GFAP.

**Table 2:** Media used for differentiation of hAMSC; Abbreviations: DMEM Dulbecco’s modified Eagles’s medium, LF low glucose, HG high glucose, FCS fetal calf serum, dexa dexamethasone, AAP ascorbic acid or ascorbat-2-phosphate, b-GP beta-glycerophosphate, MEM minimal essential medium eagle, vit D3 1alpha,25dihydroxyvitamin D3, b-ME beta-mercaptoethanol, IBMX 3-isobutyl-1-methylxanthin, IM indomethacin, ITS insulin-transferrin-selenium, TGF-b transforming growth factor-beta, BSA bovine serum albumin, BMP-2 bone morphogenetic protein-2, MCDB medium complete with trace elements, LA linoleic acid, bFGF basic fibroblast growth factor, VEGF vascular endothelial growth factor, IGF-1 insulin like growth factor-1, EGF epidermal growth factor, HGF hepatic growth factor, NEAA nonessential amino acid, IMDM Iscove’s modified DMEM, DMSO dimethyl sulfoxide, KCl potassium chloride, PDGF platelet-derived growth factor, AMP adenosine monophoshpate.

lineage	reference	media description	protocol
osteogenic	Pittenger [82]	DMEM-LG, 10% FCS, 10^−7^ M dexa, 50 μM AAP, 10 mM b-GP, 10 mM vit D3	
	In't Anker [8]	alphaMEM, 10% FCS, 10^−7^ M dexa, 50 μg/mL AAP, 5 mM b-GP	b-GP was added from day 7 on, 21 days
	Portmann-Lanz [50]	DMEM-HG, 10% FCS, 10 μmol/L dexa, 50 μg/mL AAP, 10 mol/L b-GP, 10 nmol/L vit D3	18–21 days
	Alviano [1]	DMEM, 10% FCS, 10^−8^ M dexa, 0.2 mM AAP, 10 mM b-GP	21–28 days
	Sudo [56]	DMEM-HG, 10% FCS, 10^−7^ M dexa, 0.5 μM AAP, 10 mM b-GP	28 days
	Chen [81]	DMEM, 10% FCS, 100nM dexa, 0.1 μM AAP, 10 mM b-GP, 5 × 10^−5^ M b-ME	induction on day 8 for 20 days
	Bilic [2], Diaz-Prado [4], Paracchini [18]	hMSC Mesenchymal Stem Cell Osteogenic Differentiation Medium (Lonza)	14 (Bilic) or 21 days (Diaz-Prado, Paracchini)
	Stadler [21], Wolbank [24]	MesenCult Osteogenic Stimulatory Kit (OKit, Stemcell Technologies)	21 (Wolbank) or 28 (Stadler) days
	Manochantr [57], Kang [10]	NH Osteodiff medium (Miltenyi)	14 days (Kang)
adipogenic	Pittenger [82]	medium 1: DMEM-HG, 10% FCS, 0.5 mM IBMX, 0.1 μM dexa, 10 μg/mL insulin, 100 μM IM,	medium 1: 48-72h, medium 2: 24h; 3 repeated cycles;
		medium 2; DMEM-HG, 10% FCS, 10 μg/mL insulin	28 days (Stadler)
	In't Anker [8]	alphaMEM, 10% FCS, 0.5 mM IBMX, 10^−7^ M dexa, 1.60 μM insulin, 50 μM IM, 50 μg/mL AAP	21 (In’t Anker) or 28 days (Stadler)
	Portmann-Lanz [50]	DMEM-HG, 10% FCS, 0.5 mmoL/L IBMX, 1 μmol/L dexa, 10 μmol/L insulin, 200 μmol/L IM	18–21d (Portmann-Lanz, Wolbank) or 28 days (Stadler)
	Alviano [1]	DMEM, 10% FCS, 0.5 mM IBMX, 10^−6^ M dexa, 10 μg/mL insulin, 200 μM IM	14–21 days
	Sudo [56]	medium 1: DMEM-HG, 10% FCS, 0.5 mM IBMX, 1 μM dexa, 10 ng/mL insulin, 0.2 mM IM	medium 1: 3 days, medium 2: 3 days; repeated cylces for 24–30 days
		medium 2; DMEM-HG, 10% FCS, 10 ng/mL insulin	
	Kang [10]	DMEM-LG, 10% FCS, 1mM dexa, 0.5 mM IBMX, 1 μg/mL insulin, 200 μM IM	21 days
	Diaz-Prado [4], Paracchini [18]	hMSC Mesenchymal Stem Cell Adipogenic Differentiation Medium (Lonza)	21 days
	Bilic [2], Manochantr [57]	NH Adipodiff medium (Miltenyi)	21 days
chondrogenic	Sudo [56]	alphaMEM, 3.5 g/mL glucose, 1% ITS, 100 μg/mL sodium pyruvate, 0.2 mM AAP, 10^−7^ M dexa, 10 ng/mL TGF-b3	28–30 days, pellet culture
	Alviano [1]	DMEM, 1 mM sodium pyruvate, 0.1 mM AAP, 10^−7^ M dexa, 10 ng/mL TGF-b3, 6.25 μg/mL insulin, 6.25 μg/mL transferrin, 6.25 μg/mL selenous acid, 5.33 μg/mL linolenic acid, 0.35 mM proline, 1.25 mg/mL BSA, 1/1.000 monotioglycerol	21–28 days, pellet culture
	Diaz-Prado [4]	medium 1: DMEM, 15% FCS, 5 mg/mL AAP	first 2 days medium 1,
		medium 2: DMEM, 15% knockout serum, 1 μL/mL AAP, 10 μM dexa, 1 ng/mL TGF-b3, 6 μL/mL transferrin, 10^7^ M retinoic acid	followed by 21 days medium 2; pellet culture
	Kang [10]	NH chondrogenic medium (Miltenyi)	21 days, pellet culture
	Portmann-Lanz [50]	DMEM-HG, 1% FCS, 50 ng/mL AAP, 10 ng/mL TGF-b1, 6.25 μg/mL insulin	18–21 days, monolayer
	Wei [23]	DMEM. 10% FCS, 200 ng/mL rhuBMP-2	induction 21 days after confluence, 14 days
myogenic	Portmann-Lanz [50]	DMEM-HG, 10% FCS, 50 μmol/L hydrocortison, 0.1 μmol/L dexa	18–21 days
	Alviano [1]	DMEM, 5% FCS, 40% MCDB-201, 10^−8^ M dexa, ITS-LA*BSA 1×, 10^−4^ M AAP, 10 ng/mL bFGF, 10 ng/mL VEGF, 10 ng/mL IGF-1	21 days
	Bilic [2]	DMEM-HG, 10% FCS, 5% horse serum, 50 μM hydrocortison, 0.1 μM dexa	7 days
angiogenic	Alviano [1]	DMEM, 2% FCS, 50 ng/mL VEGF	7 days, followed by matrigel assay
	König [12]	Endothelial Growth Medium-2 (Lonza) +/− 50 ng/mL VEGF	at least 10 days, followed by matrigel assay
	Kim SW [83]	Endothelial Growth Medium-2 (Lonza)	10–20 days
cardiomyogenic	Zhao [25]	DMEM, 10% FCS, 2nd d addition of 10 ng/mL bFGF or 50 ng/mL activin A	(a) 7 days after addition of growth factors; (b) coculture with rat heart explants
	Tsuji [58]	unknown	coculture with fetal murine cardiomyocytes
	Lisi [14]	DMEM, 10% FCS, 10 ng/mL EGF, 55 μM b-ME, d2-4: + 0.4 μM S-nitoso-N-acetylpenicillamine	seeded on s-IPN PRtU-PDMS/fibrin scaffold, 14 days
hepatic	Tamagawa [53]	alphaMEM, 10% FCS, 0.1 mmoL/L dexa, 20 ng/mL hHGF, 10 ng/mL hFGF, 10 ng/mL oncostatin	21 days
	Paracchini [18]	medium 1: DMEM, 10% FCS, 1% NEAA, 55 μM b-ME, 10 ng/mL EGF	first 8 days medium 1,
		medium 2: IMEM, 10% FCS, 10^−7^ M dexa, 1% NEAA, 55 μM b-ME, 10 ng/mL EGF	followed by medium 2 until day 21
pancreatic	Wei [84]	DMEM, N2 supplement, 0.1 mmoL/L nicotinamide	
	Tamagawa [22]	medium 1: DMEM-HG, 10^−6^ M retinoic acid	first 2 days medium 1, medium 2 and 3 in matrigel
		medium 2: DMEM-LG, 10% FCS, 1 × N2 supplement, 10 mM nicotinamide, 20 ng/mL hEGF	
		medium 3: DMEM-LG, 10 nM exendin-4	
	Kim J [11]	medium 1: DMEM-HG, 10% FCS, 10 mmoL nicotinamide, 4 nmol activin A, 10 nmol GLP-1	first 7 days medium 1,
		medium 2: DMEM-LG, 10% FCS, 10 mmoL nicotinamide, 4 nmol activin A, 10 nmol GLP-1	followed by 14 days medium 2
neural	Portmann-Lanz [50]	DMEM-HG, 10% FCS, 30 μmol/L all trans retinoic acid	18–21 days
	Sakuragawa [51]	DMEM, 100 μM butylated hydroxianisole, 10 μM forskolin, 2% DMSO, 5 U/mL heparin, 5 nM K252a, 25 mM KCl, 2 mM valporic acid, 1 × N2 supplement, 10 ng/mL bFGF, 10 ng/mL PDGF	
	Tamagawa [85]	medium 1: alphaMEM, 10% FCS, 1 × N2 supplement, 10 ng/mL bFGF, 10 ng/mL EGF	48 h medium 1, followed by 96 h medium 2
		medium 2: alphaMEM, 1 μM all trans retinoic acid, 200 μM butylated hydroxianisole, 1× N2 supplement, 1 mM dibutyryl cyclic AMP, 0.5 mM IBMX	followed by 96 h medium 2
	Manochantr [57]	AdvancesSTEM Neural Differentiation Medium (HyClone)	until neural like cells were observed

## 5. Differentiation potential of UC-MSC

Similar to MSC from BM and other sources, UC-derived MSC, as widely investigated by several groups, could be shown to differentiate into adipocytes [26,27,30,33,38,40,63,64,65,67,68,70,86,87,88,89,90,91,92,93,94,95,96,97,98,99,100,101,102,103,104,105,106,107,108], chondrocytes [27,29,33,38,40,63,68,70,86,87,88,89,90,91,93,94,96,98,99,103,104,106,108,109], and osteocytes [26,27,28,30,33,38,40,41,63,64,65,67,68,73,86,87,88,89,90,91,92,93,94,95,96,97,98,99,100,101,102,103,104,105,106,108,110,111,112,113,114]. An overview of the reported differentiation potential of UC-derived MSC is given in Table 3.

**Table 3:** Overview of the reported differentiation potential of UC-derived MSC

lineage	reference
osteogenic	[27,29,33,38,40,63,68,70,86,87,88,89,90,91,93,94,96,98,99,103,104,106,108,109], and osteocytes [26,27,28,30,33,38,40,41,63,64,65,67,68,73,86,87,88,89,90,91,92,93,94,95,96,97,98,99,100,101,102,103,104,105,106,108,110,111,112,113,114]
adipogenic	[26,27,30,33,38,40,63,64,65,67,68,70,86,87,88,89,90,91,92,93,94,95,96,97,98,99,100,101,102,103,104,105,106,107,108]
chondrogenic	[27,29,33,38,40,63,68,70,86,87,88,89,90,91,93,94,96,98,99,103,104,106,108,109]
myogenic	[30]
angiogenic	[86,93,101,115]
cardiomyogenic	[38,68,92,96,116，^117^，^118^]
hepatic	[109,119,120]
pancreatic	[121，^122^，^123^，^124^]
neural	[31,32,33,35,36,65,68,91,104,125,126,127,128]

Besides the “classical” mesenchymal lineages differentiation of UC-MSC into cells of all three germ layers has been described. Thus it has been shown that UC-derived MSC could be differentiated into functional endothelial progenitor cells after induction with VEGF and bFGF [93,101,115]. Furthermore, vessel-like structure formation and even differentiation into skeletal myocyte-like cells could be observed [30,86,93].

For the cardiomyogenic differentiation of UC-MSC 5-azacytidine was shown to be a potent agent to induce differentiation into cardiomyocyte-like cells shown by changing morphology to a typical cardiac phenotype and the expression of specific cardiac markers like N-cadherin, cardiac troponin, desmin, alpha sarcomeric actin, and myosin heavy chain [38,68,92,116,117]. Additionally Sphingosine-1-phosphate (S1P) was shown to induce cardiomyogenic differentiation of UC-derived MSC, as well [118]. In response to S1P UC-MSC displayed a cardiomyocyte-like morphology and expressed alpha-actinin and myosin heavy chain proteins. Furthermore, differentiated cells displayed a cardimyocyte-like action potential and voltage gated currents when patch clamping recording was applied. However, other groups could not detect cardiac marker expression after induction of cardiogenic differentiation of UC-MSC and to our knowledge, only one group reported slightly spontaneous beating of differentiated cells after 21 days of culture [96,116].

Studies by several groups indicate that UC-derived MSC can also differentiate into cells of the endodermal lineage. Thus it could be shown that UC-MSC, when applying appropriate culture conditions, can differentiate into hepatocyte-like cells [109,119,120]. After induction with several growth factors, like hepatocyte growth factor (HGF), bFGF, or FGF-4 cells displayed a hepatocyte-like morphology and expressed several hepatic markers like albumin, alpha-fetoprotein, cytokeratin-19, connexin-32, and dipeptidyl peptidase IV. Furthermore, differentiated cells displayed typical hepatocyte-specific functions, including albumin secretion, urea uptake, glycogen storage, and low-density lipoprotein uptake. Noteworthy first results indicate that UC-MSC do not lose their *in vitro* immune privilege after differentiation underlining that the UC-tissue is a favorable source of MSC for clinical applications [120]. However, it should be mentioned that some authors pointed out that they did not observe some important characteristics of functional liver cells in hepatic differentiated UC-MSC suggesting that these cells can only be differentiated into immature hepatocytes [119]. 

Regarding other cell types of the endodermal lineage UC-MSC could also be shown to form islet-like cell clusters secreting insulin in response to glucose challenge and expressing pancreatic beta-cell development-related genes like PDX-1 after induction with appropriate agents like nicotinamide or beta-mercaptoethanol [121,122,123,124]. Additionally it could be shown that differentiated insulin-producing UC-derived cells could alleviate hyperglycemia in diabetic mice [122]. 

Investigating the potential of UC-derived MSC to differentiate into cells of the ectodermal lineage several groups reported differentiation into neural-like cells [31,32,33,35,36,65,68,91,104,125,126,127,128]. Upon stimulation with potent agents like b-FGF, or retionic acid morphological changes and neuro-specific marker expression, like nestin, β-tubulin III, or neurofilament M, could be observed on the mRNA as well as protein level. 

The published data gives strong evidence that UC-derived MSC are mulitpotent cells which are capable of differentiating into cells of all three germ layers. However, since UC-MSC are a rather heterogenic population, especially when isolated from whole UC-tissue or the WJ, it still remains to be investigated whether all or only cells from a distinct compartment of the cord are capable of differentiating into all cell types mentioned above. For instance Sarugaser *et al.* showed that perivascular-derived UC-cells displayed a high osteogenic differentiation potential, but could not be differentiated into neuron-like cells [41]. In contrast Suzdal’tseva *et al.* reported an only poor osteogenic potential of UC vein subendothelial tissue-derived cells [99] and Karahuseyinoglu *et al.* could identify sub-populations in WJ-derived cell cultures from which one, predominately expressing cytokeratin, could not be differentiated into neuron-like cells [33].

## 6. Immunological properties of hAMSC and UC-MSC

Besides their multi-lineage differentiation potential, MSC were also found to be only weakly immunogenic and exhibit immunomodulatory properties which means that they escape immunological defense mechanisms and are able to suppress several functions of immunocompetent cells. It has been reported that hAMSC and UC-MSC possess an immunoprivileged status as well, which is in part due to the expression of low to moderate levels of surface MHC-I, and the presence of low or even absent levels of MHC II and costimulatory molecules (e.g., CD40, CD80, CD86) [6,8,11,19,55,65,129,130,131].

Implantation of hAMSC into animals resulted in successful and persistent engraftment in multiple organs and tissues. hAMSC survived for at least two months in xenotransplanted myocardial infarcts in rat hearts, showing low immunogenicity [25]. Tsuji *et al.* [58] found hAMSC transdifferentiated towards the cardiomyogenic lineage surviving for more than 4 weeks after implantation into infracted myocardium in non-immunosuppressed rats. Similarly, implantation of hAMSC into the subfacial space of the abdominal muscle in mice and intraperitoneal or intravenous injection into neonatal swine did not lead to transplant rejection during the observed experimental period of 35 and 61 days, respectively [23,132]. 

Implantation of porcine UC-MSC into rat brains resulted in engraftment and proliferation of the cells for up to eight weeks without immune rejection or the formation of teratomas [133]. Furthermore, when human UC-derived MSC were transplanted into Parkinson’s disease (PD) model rats, the cells did not induce the formation of brain tumors or immune rejections but mitigated induced motor deficits [39]. Further studies could reveal survival and proliferation of xenotransplanted UC-derived cells for five and eight weeks, respectively [134,135]. 

*In vitro*, immunological tolerance was demonstrated by low or absent allogenic reaction, coculturing unstimulated allogenic peripheral blood mononuclear cells (PBMC) or other immune cells and hAMSC [49,55] and UC-MSC [129,130,136,137], respectively.

Various *in vitro* studies have been conducted to define the immunomodulatory potential of MSC and the underlying mechanisms are still not fully understood. In this context it is still under discussion whether the regulatory effects depend on cell-to-cell contacts or are mediated by soluble factors. Several groups have used *in vitro* co-culture setups to analyze the inhibitory effects of hAMSC and UC-MSC on the proliferation of PBMC or other immunocompetent cells stimulated by mixed lymphocyte reaction or mitogens (e.g., phytohemagglutinin (PHA)) which was found to be inhibited by hAMSC and UC-MSC in a dose-, cell-contact-dependent as well as factor-mediated manner [129,130,131,136,137,138,139,140]. Interestingly cryopreservation of hAMSC significantly decreased the immunomodulatory potential, which could, however, not be linked to changes in the expression of MHC-I and -II [55]. With regard to soluble factors, several groups have reported that MSC constitutively or upon stimulation secrete a variety of mediators among which prostaglandin E2 (PGE2), indoleamine 2,3-dioxygenase (IDO) and nitric oxide (NO) seem to be key molecules in immune regulation. Chen *et al.* used transwell co-cultures of stimulated PBMC with UC-MSC to show that immunosuppression was mainly mediated through a PGE2-dependent mechanism since PGE2-production was enhanced upon co-cultivation and blocking of PEG2 biosynthesis completely abolished immunosuppressive effects [129]. Furthermore, studies by Cutler *et al.* implicate monocytes as a key intermediary in UC-MSC–induced suppression of T cell proliferation since function and the allostimulatory capacity of monocytes are downregulated, which is in part mediated by UC-MSC-derived PGE2 [141].

Kang *et al.* confirmed the inhibitory effect of hAMSC on mitogen-stimulated PBMC [10]. IFNgamma and IL-17 produced by PBMC was shown to decrease, whereas interleukin (IL)-10 increased. Concomitantly hAMSC increased levels of secreted TGF-b, HGF, PGE_2_ and IDO. Pretreatment of hAMSC with IFNgamma was shown to enhance their anti-proliferative effects on stimulated PBMC and T cells [13]. Whereas CD105 and surface density of CD90 decreased, CD54, HLA-DR, CD40, as well as the inhibitory co-stimulatory molecules PD-L1 and PD-L2 were upregulated. Coculturing PBMC and IFNgamma-treated hAMSC revealed downregulation of chemokines (RANTES, IP-10, MIG, MIP-1alpha, MIP-1beta, MCP-1), cytokines (IL-21, IL-12p70, IL-9, IFNgamma, IL-13, TNFalpha, IL-17A, IL-12/IL23p40, IL-4, IL-10) and sFAS-L as well as sCD40L, whereas IL-11, IL-6, IL-8 and LIF were upregulated. Quite similar results were obtained when analyzing the secretome of stimulated T cell in presence of hAMSC (except for MCP-1 and IP-10 which were increased). Two subpopulations of hAMSC have been described which differently express MHC-II, CD45, CD14 and CD86, thus resulting in both suppressive and stimulatory properties [49]. The stimulatory effect on anti-CD3-primed T cells at low concentrations was reported to be attributed to the HLA-DR positive subpopulation. These cells were shown to have characteristics similar to human monocytes but have proven to be of fetal origin.

Besides T cells, MSC were found to influence several other immune cells. Che *et al.* could show that UC-MSC significantly suppressed the proliferation, differentiation, and immunoglobulin secretion of B cells *in vitro* [142]. Furthermore, hAMSC have been shown to suppress differentiation and maturation of monocytes into dendritic cells (DC), arresting them in the G0 phase of the cell cycle. The presence of hAMSC inhibited the production of inflammatory cytokines including TNFalpha, CXCL10, CXCL9 and CCL5 in co-cultured monocytes [15]. Taken together these effects resulted in a diminished capacity of DC to activate T cells. Moreover, a comparison of hAMSC to human adipose tissue derived stem cells [143] demonstrated superiority of hAMSC regarding the inhibition of stimulated monocytes to differentiate into DC. In the described cell contact independent co-culture setting, high levels of PGE_2_ and HGF were found. However, this superiority of hAMSC might not be related merely to their early developmental stage, since bone marrow derived cells similarly suppress DC differentiation [144,145,146], whereas multipotent cord blood derived cells do not share these properties [147].

Moreover, Tsuji *et al.* [58] not only found surviving hAMSC transdifferentiated towards the cardiomyogenic lineage at least 4 weeks after implantation into rat infracted myocardium, they also provide an *in vivo* study on the immunomodulation [58]. They found that retreatment of hAMSC with the anti-inflammatory cytokine IL-10 increased the level of HLA-G expressed on hAMSC, which may play a role in initial processes of tolerance. Furthermore, regulatory T cells, defined as FOXP3 positive lymphocytes, were detected adjacent to implanted hAMSC into infracted myocardium of rat hearts, which may be involved in maintenance of tolerance.

## 7. Immortalization of hAMSC and UC-MSC

Applying MSC for cell therapy, higher cell numbers are required. Furthermore, well characterized cell lines, constantly growing while maintaining their typical properties, are highly valuable for research. Immortalization is one opportunity to fulfill this requirement. Wolbank *et al.* [24] could successfully establish two cell lines derived from hAMSC using a retroviral transfection system for introduction of hTERT. hTERT overexpression in hAMSC maintained fibroblastic morphology, surface expression of most hematopoietic and mesenchymal stem cell marker as well as adipogenic and osteogenic differentiation potential. Furthermore, immunological properties were similar to parental cells and no chromosomal abnormalities or tumorigenic conversion was observed.

As recently reported, also immortalized MSC from UC vein and Wharton’s jelly have been established [148,149]. First analysis of these UC-MSC expressing ectopic hTERT revealed that the findings regarding immortalized hMSC from common tissues like bone marrow and amnion are comparable to preliminary data collected from immortalized UC-MSC. The modified cells increased their population doubling capacity up to more than 100 PD with no morphological or karyotypic alterations [149]. Flow cytometric analysis of the surface molecules revealed expression of MSC specific markers (CD13, CD29, CD105) and lack the expression of CD 34, CD45, HLA-DR. Furthermore, these cells were proven to express a set of genes like Oct-4, Nanog, ZFX, Bmi-1 and Nucleostemin which are known to be associated with their self-renewal capacity [148]. Immunologic analysis show maintenance of the MSC immunotolerance as well as the absence of any tumorigenic conversion *in vitro* or in nude mice during a period of 2 weeks [149]. Furthermore, the differentiation of immortalized UC-MSC in hepatocyte-like cells and subsequent analysis of liver specific markers showed that these MSC also maintain their multipotent differentiation potential similar to the isolated parental cells in early passages.

However, less is known about the influence of such modifications to the entire expression patterns and the impact on hMSC behavior. Such suggestions based on the fact that immortalization via common transfection methods based on randomized insertion of the hTERT gene into the genome of hMSC. Although immortalized hMSC exhibit near limitless potential, considering the application of such cells in clinical trials require either the localization of the exact positions of the transgenes or transfection methods which allow exact gene targeting.

## 8. Prospective Clinical Applications: Current State and Outlook

Isolated from biologic waste, ethically non-problematic, stem cells from full-term umbilical cord tissue and amniotic membrane are easily available and may present an attractive completion to other classically established stem cells (e.g., from bone marrow) in different clinical approaches. Since it has been shown in numerous papers that MSC have the ability to down-regulate immune response and support tissue repair mechanisms their use has been widespread for the treatment of many different diseases. Currently performed studies often apply MSC from bone marrow (39 studies in different stages, several involving a combination therapy with drugs). Over the last decade’s 125 clinical trials has been documented using MSC from bone marrow, 43 studies for UC-MSC (3 for MSC from Wharton’s jelly respectively) and only one is documented for MSC isolated from amniotic membrane [150]. 

The application of UC-MSC in the documented studies include treatment of Graft *vs.* Host Disease (GvHD), hepatic cirrhosis, colitis ulcerosa, type 1 diabetes and treatment of diabetic foot wounds, cardiomyopathy, Alzheimer disease, different autoimmune diseases e.g., multiple sclerosis and muscular dystrophy, neuromyelitis optica and rheumatoid arthritis. 

UC-MSC have for instance been applied in the treatment of 160 patients after myocardial infarction in a double-blind, placebo-controlled, multicenter phase 2 trial (by Navy General Hospital, Beijing, completed July 2012) [151]. Several papers report on differentiation of UC-MSC into cardiomyocyte-like cells [38,152,153], but functionality of these derived cells is also controversially discussed [96,154].

Twenty-two clinical trials use MSC for the treatment of Graft *vs.* Host Disease (GvHD) whereas the most recent studies started recruiting patients in 2012. UC-MSC have also been applied for the treatment of Alzheimer disease (NCT01547689) and in HIV patients (NCT01213186). Successful and promising preclinical studies on UC-MSC and hAMSC performed to date and their diverse properties offer these cells the possibility for future clinical use in the treatment of various diseases. Till then some major obstacles, such as translation from research to GMP-scale, market authorization and clinical application will need to be resolved.

## 9. Conclusion

Taken together, the results of published data and clinical trials on MSC have proven to provide great potential for different therapeutic applications. Due to their young age, UC-MSC and hAMSC possess high proliferative capacity and expansion potential; thus, the *in vitro* expansion process can be reduced with regard to time and passage number. In addition, a reduction of labor intensive work risks of contamination and damages (e.g., inadvertant epigentic modifications) during *in vitro* expansion can be achieved. The combination of retaining multilineage differentiation potential with their immunmodulatory properties make MSC from umbilical cord tissue and amnion promising cell therapeutics. 
